# Supplementary material for: Comparative functional survival and equivalent annual cost of 3 long-lasting insecticidal net (LLIN) products in Tanzania: A randomised trial with 3-year follow up
Source: PLoS Med. 2020 Sep 18;17(9):e1003248. doi: 10.1371/journal.pmed.1003248 (PMC7500675; doi:10.1371/journal.pmed.1003248)

**S11 Fig**

**The location of damage on nets by year after distribution and net brand measured by proportionate hole index.** The location of damage was measured by dividing the nets into 4 zones of approximately 37.5 cm when counting holes. The majority of damage is concentrated in the bottom quarter of the net. In questionnaire surveys, 94% of users reported that they tucked their nets under a mattress or reed mat and it is indeed the act of tucking the net that causes damage to this bottom quadrant. Tucking the net makes this damaged area unavailable to mosquitoes but makes the user perceive the net as unserviceable.

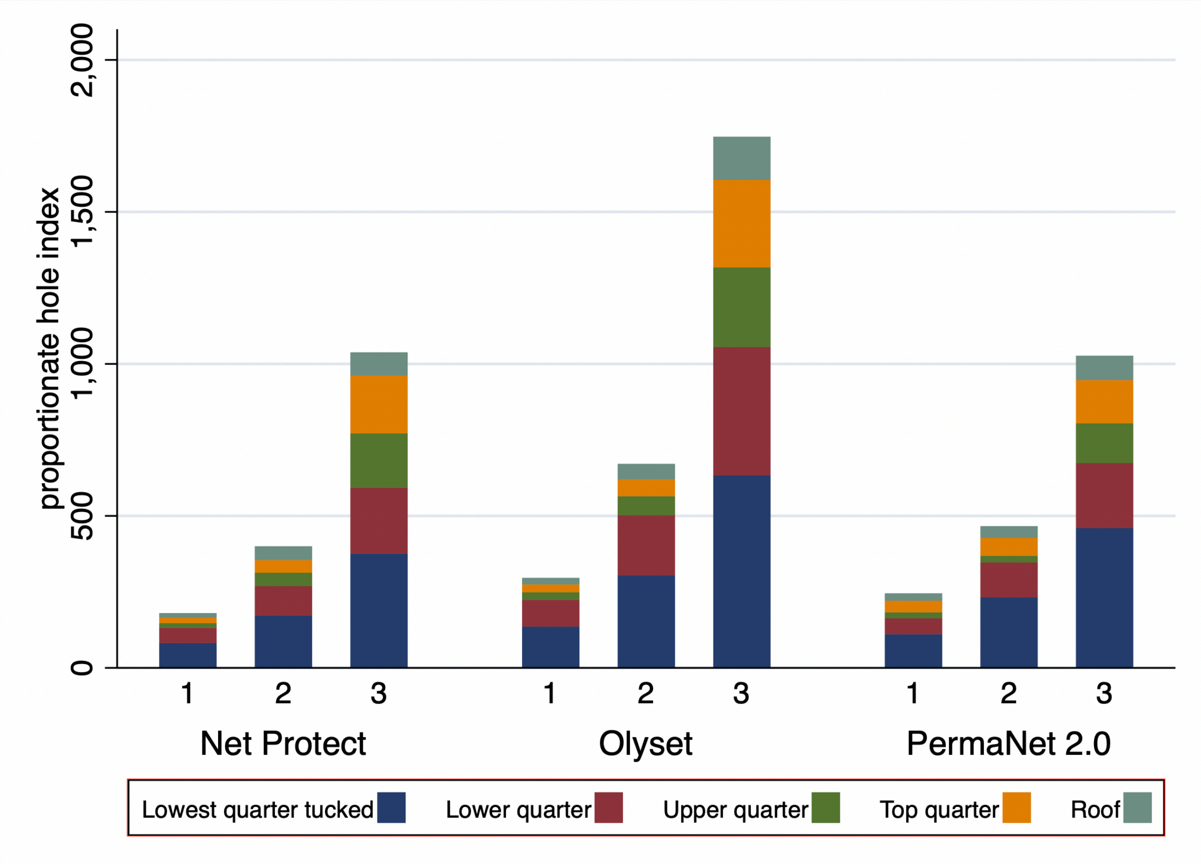

Supplement: S2 Fig — (PDF) [file pmed.1003248.s002.pdf]
