# Supplementary material for: Comparative functional survival and equivalent annual cost of 3 long-lasting insecticidal net (LLIN) products in Tanzania: A randomised trial with 3-year follow up
Source: PLoS Med. 2020 Sep 18;17(9):e1003248. doi: 10.1371/journal.pmed.1003248 (PMC7500675; doi:10.1371/journal.pmed.1003248)
Supplement: S1 STROBE Checklist — (PDF) [file pmed.1003248.s003.pdf]

S12 STROBE Checklist

	Item No	Recommendation	Location / line
**Title and abstract**	1	(*a*) Indicate the study's design with a commonly used term in the title or the abstract	Title
		(*b*) Provide in the abstract an informative and balanced summary of what was done and what was found	Abstract
**Introduction**			
Background/rationale	2	Explain the scientific background and rationale for the investigation being reported	Introduction
Objectives	3	State specific objectives, including any prespecified hypotheses	Abstract background<br>Introduction paragraph 6<br>Table 1
**Methods**			
Study design	4	Present key elements of study design early in the paper	Introduction paragraph 5
Setting	5	Describe the setting, locations, and relevant dates, including periods of recruitment, exposure, follow-up, and data collection	Figure 1<br>Methods paragraph 1<br>S1 Table
Participants	6	(*a*) *Cohort study*—Give the eligibility criteria, and the sources and methods of selection of participants. Describe methods of follow-up	Cohort study<br>Methods paragraph 1<br>Follow up<br>Methods paragraph 2 and 3
		(*b*) *Cohort study*—For matched studies, give matching criteria and number of exposed and unexposed	NA
Variables	7	Clearly define all outcomes, exposures, predictors, potential confounders, and effect modifiers. Give diagnostic criteria, if applicable	Table 1
Data sources/ measurement	8*	For each variable of interest, give sources of data and details of methods of assessment (measurement). Describe comparability of assessment methods if there is more than one group	Table 1
Bias	9	Describe any efforts to address potential sources of bias	Blinding Methods Paragraph 1<br>Randomisation Methods Paragraph 1<br>Range of settings Methods Paragraph 1, Figure 1
Study size	10	Explain how the study size was arrived at	Lorenz et al 2014 [21]
Quantitative variables	11	Explain how quantitative variables were handled in the analyses. If applicable, describe which groupings were chosen and why	Methods statistical analysis
Statistical methods	12	(*a*) Describe all statistical methods, including those used to control for confounding	Methods statistical analysis
		(*b*) Describe any methods used to examine subgroups and interactions	Clustering at village level: Methods statistical analysis<br>Balance between arms: Table 2
		(*c*) Explain how missing data were addressed	Electronic data capture minimised missing data and data errors: Methods paragraph 2
		(*d*) *Cohort study*—If applicable, explain how loss to follow-up was addressed	Sample size was adequate as it was calculated on a 50% loss to follow up greater than that which was observed in the study Ref [21]
		(*e*) Describe any sensitivity analyses	N/A
**Results**			**Location / line**
Participants	13*	(a) Report numbers of individuals at each stage of study—eg numbers potentially eligible, examined for eligibility, confirmed eligible, included in the study, completing follow-up, and analysed	S1 Table study flow<br>S4 Table functional survival<br>S5 Table attrition
		(b) Give reasons for non-participation at each stage	NA
		(c) Consider use of a flow diagram	S1 Table
Descriptive data	14*	(a) Give characteristics of study participants (eg demographic, clinical, social) and information on exposures and potential confounders	Table 2
		(b) Indicate number of participants with missing data for each variable of interest	S1 Table study flow<br>S4 Table functional survival<br>S5 Table attrition
		(c) Cohort study—Summarise follow-up time (eg, average and total amount)	S4 Table functional survival
Outcome data	15*	Cohort study—Report numbers of outcome events or summary measures over time	S4 Table functional survival<br>S5 Table attrition
Main results	16	(a) Give unadjusted estimates and, if applicable, confounder-adjusted estimates and their precision (eg, 95% confidence interval). Make clear which confounders were adjusted for and why they were included	Results
		(b) Report category boundaries when continuous variables were categorized	N/A
		(c) If relevant, consider translating estimates of relative risk into absolute risk for a meaningful time period	N/A
Other analyses	17	Report other analyses done—eg analyses of subgroups and interactions, and sensitivity analyses	N/A
**Discussion**			
Key results	18	Summarise key results with reference to study objectives	Results
Limitations	19	Discuss limitations of the study, taking into account sources of potential bias or imprecision. Discuss both direction and magnitude of any potential bias	Discussion paragraph 5 & 8
Interpretation	20	Give a cautious overall interpretation of results considering objectives, limitations, multiplicity of analyses, results from similar studies, and other relevant evidence	Results
Generalisability	21	Discuss the generalisability (external validity) of the study results	Discussion
Other information			
Funding	22	Give the source of funding and the role of the funders for the present study and, if applicable, for the original study on which the present article is based	Funding
