# Supplementary material for: Comparative functional survival and equivalent annual cost of 3 long-lasting insecticidal net (LLIN) products in Tanzania: A randomised trial with 3-year follow up
Source: PLoS Med. 2020 Sep 18;17(9):e1003248. doi: 10.1371/journal.pmed.1003248 (PMC7500675; doi:10.1371/journal.pmed.1003248)
Supplement: S1 Table — The number of interviews completed each year, loss to follow-up, and the number of study nets evaluated for each durability component is shown. (PDF) [file pmed.1003248.s004.pdf]

S1 Table

Study Flow. The number of interviews completed each year, loss to follow up and the number of study nets evaluated for each durability component is shown.

	2014 (Year 1)	2015 (Year 2)	2016 (Year 3)
**Households interviewed**	**87.2% (2,962/3,398)**	**95.7% (2,834/2,962)**	**96.3% (2,730/2,834)**
**Household loss to follow up**	**12.8% (436/3,398)**	**4.3% (128/2,962)**	**3.7% (104/2,834)**
**Study nets loss to follow up**	**8.3% (880/10,598)**	**7.2% (539/7,477)**	**10.4% (551/5,311)**
**Nets lost from households**	**23.1% (2,241/9,726)**	**23.5% (1,627/6,938)**	**39.0% (1,855/4,760)**
**Nets inspected for holes**	**82.5% (6,166/7,477)**	**90.1% (4,783/5,311)**	**86.7% (2,519/2,905)**
**Nets evaluated by Ifakara Ambient Chamber Test**	**142**	**144**	**140**
**Nets evaluated for bio efficacy**	**142**	**144**	**140**
**Nets evaluated for chemical content**	**144**	**144**	**144**
