# Supplementary material for: Comparative functional survival and equivalent annual cost of 3 long-lasting insecticidal net (LLIN) products in Tanzania: A randomised trial with 3-year follow up
Source: PLoS Med. 2020 Sep 18;17(9):e1003248. doi: 10.1371/journal.pmed.1003248 (PMC7500675; doi:10.1371/journal.pmed.1003248)
Supplement: S2 Table — (PDF) [file pmed.1003248.s005.pdf]

S2 Table
Parameters used in simulation of lifetimes for equivalent annual cost simulation analysis

Parameter	Distribution for simulation	Parameter 1	Parameter 2
Lambda (base hazard)	Lognormal	Mean log= -3·874	SD log = -11·393
Proportional Hazard NetProtect	Lognormal	Mean log = -0·373	SD log = -7·806
Proportional Hazard PermaNet	Lognormal	Mean log = -0·304	SD log = -7·704
Scale of Weibull Olyset	Weibull	V = 1·08	
Scale of Weibull NetProtect	Weibull	V = 1·11	
Scale of Weibull PermaNet	Weibull	V = 1·12	
