# Supplementary material for: Comparative functional survival and equivalent annual cost of 3 long-lasting insecticidal net (LLIN) products in Tanzania: A randomised trial with 3-year follow up
Source: PLoS Med. 2020 Sep 18;17(9):e1003248. doi: 10.1371/journal.pmed.1003248 (PMC7500675; doi:10.1371/journal.pmed.1003248)
Supplement: S3 Table — Data represent numbers of respondents (percent) reporting use of nets. (PDF) [file pmed.1003248.s006.pdf]

S3 Table

Reported net use the previous night by net product and time point. Data represent numbers of respondents (percent) reporting use of nets.

	Olyset	PermaNet	NetProtect	Total	p-value
**10 months**					
**not used**	440 (20·4)	410 (18·6)	448 (20·8)	1298 (19·9)	P = 0·195
**used**	1714 (79·6)	1799 (81·4)	1705 (79·2)	5218 (80·1)	
**total**	2154	2209	2153	6516	
**22 months**					
**not used**	514 (32.2)	518 (29·8)	538 (32·4)	1570 (31·4)	P = 0·648
**used**	1082 (67.8)	1221 (70·2)	1122 (67·6)	3425 (68·6)	
**total**	1596	1739	1660	4995	
**36 months**					
**not used**	358 (49·3)	449 (45·2)	471 (52·5)	1278 (48·8)	P = 0·189
**used**	368 (50·7)	545 (54·8)	426 (47·5)	1339 (51·2)	
**total**	726	994	897	2617	
