# Supplementary material for: Comparative functional survival and equivalent annual cost of 3 long-lasting insecticidal net (LLIN) products in Tanzania: A randomised trial with 3-year follow up
Source: PLoS Med. 2020 Sep 18;17(9):e1003248. doi: 10.1371/journal.pmed.1003248 (PMC7500675; doi:10.1371/journal.pmed.1003248)
Supplement: S4 Table — (PDF) [file pmed.1003248.s007.pdf]

S4 Table
Number at risk (functional survival)

	Time point (months)	At risk	censored	Failed
**Olyset**	10	3520	1019	451
	22	2050	399	554
	36	1097	307	398
**PermaNet**	10	3513	1002	314
	22	2197	406	470
	36	1321	331	404
**NetProtect**	10	3538	1020	307
	22	2211	407	436
	36	1368	372	403
