# Supplementary material for: Comparative functional survival and equivalent annual cost of 3 long-lasting insecticidal net (LLIN) products in Tanzania: A randomised trial with 3-year follow up
Source: PLoS Med. 2020 Sep 18;17(9):e1003248. doi: 10.1371/journal.pmed.1003248 (PMC7500675; doi:10.1371/journal.pmed.1003248)
Supplement: S5 Table — (PDF) [file pmed.1003248.s008.pdf]

S5 Table
Number at risk (attrition)

	Time point (months)	At risk	censored	Failed
**Olyset**	10	3520	741	187
	22	2592	501	404
	36	1687	406	510
**PermaNet 2.0**	10	3513	759	132
	22	2622	444	351
	36	1827	439	383
**NetProtect**	10	3538	758	163
	22	2617	504	367
	36	1746	412	413
