# Supplementary material for: Comparative functional survival and equivalent annual cost of 3 long-lasting insecticidal net (LLIN) products in Tanzania: A randomised trial with 3-year follow up
Source: PLoS Med. 2020 Sep 18;17(9):e1003248. doi: 10.1371/journal.pmed.1003248 (PMC7500675; doi:10.1371/journal.pmed.1003248)
Supplement: S6 Table — (PDF) [file pmed.1003248.s009.pdf]

S6 Table

Median hole surface area in cm^2 and interquartile range (IQR) by net product and time point

		**Olyset**	**PermaNet**	**NetProtect**	**Total**	**p-value**
**Median hole surface area in cm^2 (IQR)**	10 months	38 (0 - 308)	6 (0 - 145)	8 (0 - 94)	19 (0 - 182)	P < 0·001
	22 months	247 (13 - 969)	84 (0 - 614)	61 (0 - 364)	97 (1 – 669)	P < 0·001
	36 months	459 (66 - 1708)	295 (29 - 1220)	152 (13 - 838)	277 (31 - 1185)	P < 0·001
