# Supplementary material for: Comparative functional survival and equivalent annual cost of 3 long-lasting insecticidal net (LLIN) products in Tanzania: A randomised trial with 3-year follow up
Source: PLoS Med. 2020 Sep 18;17(9):e1003248. doi: 10.1371/journal.pmed.1003248 (PMC7500675; doi:10.1371/journal.pmed.1003248)
Supplement: S7 Table — (PDF) [file pmed.1003248.s010.pdf]

S7 Table

I-ACT (Ifakara Ambient Chamber Test) results on mosquito mortality and blood feeding inhibition by net product and time point in months. Data from the I-ACT test were analysed by binary logistic regression and the Odds Ratio (OR) and 95% confidence interval (95% CI) of the Odds Ratio are shown with Olyset as the reference net; percentages of net products meeting optimal WHO bio efficacy criteria in the I-ACT by time point in months. 95% confidence intervals in parenthesis and compared with a χ^2 test. Numbers passing / numbers tested in square brackets [n/N]†.

	% (n/N) Mosquito Mortality			% (n/N) Mosquito Blood feeding Inhibition			% (CI) [n/N] Nets meeting WHO bio efficacy criteria		
**Months**	**10**	**22**	**36**	**10**	**22**	**36**	**10**	**22**	**36**
**Olyset**	87% (2355/2700)	69% (1895/2880)	70% (2009/2880)	99.7% (2691/2700)	98.1% (2826/2880)	93.1% (2682/2880)	100% (92,100) [49/49]	96% (86,99) [46/48]	88% (75,95) [42/48]
**OR (95% CI)**	1	1	1	1	1	1			
**PermaNet**	98% (2686/2730)	91% (2635/2880)	88% (2530/2880)	99.6% (2719/2730)	98.8% (2845/2880)	96.5% (2779/2880)	100% (93, 100) [48/48]	98% (89,100) [47/48]	96% (86,99) [46/48]
**OR (95% CI)**	6·4 (3·73-11·11)	4·09 (2·77-6·04)	2·16 (1·21-3·85)	1·50 (0·36-6·22)	1·01 (0·44-2·34)	1·88 (0·88-3·99)			
**NetProtect**	99% (2705/2730)	96% (2764/2880)	94.1% (2711/2880)	99.9% (2726/2730)	99.9% (2876/2880)	94.1% (2711/2880)	100% (92, 100) [47,47]	100% (92,100) [48/48]	92% (80,98) [44/48]
**OR (95% CI)**	14·7 (7·87-27·34)	12·82 (7·52-21·86)	3·43 (2·33-5·06)	1·87 (0·40-8·84)	12·70 (1·97-81·75)	0·94 (0·48-1·82)			
**p-value***	<0·001	<0·001	<0·001	0·697	0·026	0·200	-	0·360	0·336

† WHO optimal insecticide effectiveness criteria of ≥80% 24-hour mortality or ≥90% blood feeding inhibition were used.
*p-value for the comparison between the three nets. The p-values for differences in mosquito mortality between PermaNet and NetProtect were 0.024, <0.001, and 0.143 at 10, 22 and 36 months, respectively.
