# Supplementary material for: Comparative functional survival and equivalent annual cost of 3 long-lasting insecticidal net (LLIN) products in Tanzania: A randomised trial with 3-year follow up
Source: PLoS Med. 2020 Sep 18;17(9):e1003248. doi: 10.1371/journal.pmed.1003248 (PMC7500675; doi:10.1371/journal.pmed.1003248)
Supplement: S8 Table — (PDF) [file pmed.1003248.s011.pdf]

S8 Table

Number of nets, mean active ingredient (AI) content (g/kg), range (g/kg) and between net variation (%RSD), percentage of active ingredient lost over time, mean *R*-alpha isomer content (g/kg) and percentage of deltamethrin (only for PermaNet 2.0 and NetProtect), in net samples at baseline and three follow up time points.

LLIN	Test	Months after distribution			
		0	10	22	36
**Olyset**	**Number**	10	49	48	48
	**AI content (mean)**	20·3	16·2	14·8	13·0
	**AI content (range)**	20·0 – 20·9	8·4 – 19·3	7·5 – 20·0	3·3 – 19·8
	**AI variation (RSD)**	1%	13%	19%	33%
	**AI lost**	-	20%	27%	36%
**PermaNet 2.0**	**Number**	10	48	48	48
	**AI content (mean)**	1·45	0·75	0·47	0·40
	**AI content (range)**	1·36 – 1·68	0·03 – 1·83	< 0·01 – 1·51	< 0·01 – 1·55
	**AI variation (RSD)**	7%	58%	77%	106%
	**AI lost**	-	48%	68%	72%
	***R*-alpha content (mean)**	0·02	< 0·01	< 0·01	< 0·01
	***R*-alpha (% of deltamethrin)**	1·4%	< 1·3%	< 1·3%	< 1·3%
**NetProtect**	**Number**	10	47	48	48
	**AI content (mean)**	1·35	0·91	0·52	0·40
	**AI content (range)**	1·26 – 1·43	0·07 – 1·88	0·19 – 1·24	0·05 – 0·99
	**AI variation (RSD)**	4%	32%	42%	60%
	**AI lost**	-	33%	61%	70%
	***R*-alpha content (mean)**	0·35	0·29	0·25	0·20
	***R*-alpha (% of deltamethrin)**	26%	32%	48%	50%
