# Supplementary material for: Comparative functional survival and equivalent annual cost of 3 long-lasting insecticidal net (LLIN) products in Tanzania: A randomised trial with 3-year follow up
Source: PLoS Med. 2020 Sep 18;17(9):e1003248. doi: 10.1371/journal.pmed.1003248 (PMC7500675; doi:10.1371/journal.pmed.1003248)
Supplement: S1 Text — (PDF) [file pmed.1003248.s012.pdf]

S9 Text

Literature review on durability of PermaNet 2.0, Olyset and NetProtect

A systematic literature review of published research findings was carried out using Pub-Med on 19th April 2020. Articles were identified and screened if they included any combination of the search terms in the title or abstract. Additional articles were identified through a hand search of all references in articles identified through the initial keyword search. The review was limited to English language articles published between January 1, 1990 and April 19th, 2020. Search terms were itn durability OR llin durability OR bednet durability AND olyset OR permanet OR netprotect. In addition, World Health Organization Pesticide Evaluation Scheme (WHOPES) reports that were used to give the three net brands WHOPES recommendation (PQ listing) were accessed from the WHOPES website[1].

A total of 129 references were located and the abstracts were screened. Twenty-five full text publications met initial inclusion criteria and the full texts were further screened. Inclusion criteria were: 1) one of the three net brands were evaluated in the study; 2) nets were evaluated after operational use; 3) at least one of the following criteria were evaluated a) attrition, b) bio-efficacy, c) chemical content, d) physical integrity, e) functional survival; 5) nets were evaluated using WHO methodology. Twenty-three published papers and 4 WHOPES reports were included in the final review (Table S1). An average proportionate hole index (pHI) or holed surface area and average proportion of nets passing WHO bio efficacy criteria was calculated from those trials that were conducted for three years and compared to the results of the trial presented in this publication.

PermaNet 2.0

***WHO* recommendation:** PermaNet 2.0 was granted World Health Organization (WHO) recommendation as a long lasting insecticidal net (LLIN) in 2008 based on a multi-centre study in six countries [1]. Data were highly variable, but when bio-efficacy data was pooled from all countries, the overall proportion of nets passing was greater than 80%. In the original report only hole sizes are given. Therefore, after converting the data in the report to the pHI using [2] resulted in a mean pHI of 389 (0-830), thus the majority of the nets were in "serviceable" condition after 3 years.

***Review of all available* data:** Including data from peer-reviewed publications in addition to the WHO sponsored studies (N=8), pooled proportion of nets passing bio efficacy criteria at 36 months was 84% and mean (95% CI) pHI was 380 (90-667).

Olyset

***WHO* recommendation:** Olyset was given LLIN status based on pooled data from eight African countries [3]. The multi-country survey was not designed to measure the longevity of Olyset Nets (i.e. the proportion of nets that remain in domestic use among those distributed at the beginning), and the report acknowledges that the retrospective design did not capture the possibility that worn-out nets were discarded by the owners. Converting the data in the report to pHI using [2] resulted in a mean pHI of 823 (95% CI 416-1,231) in year three, thus the majority of nets are in "too torn" condition after 3 years. Using combined cone and tunnel test data, 71% of nets passed bio-efficacy criteria.

***Review of all available data*:** Including data from peer reviewed publications in addition to the WHO sponsored studies (N=10), pooled proportion of nets passing bio efficacy criteria at 36 months was 84% and mean (95% CI) pHI was 901 (143-1,658).

NetProtect

[1] https://www.who.int/whopes/resources/meeting_reports/en/

WHO recommendation
NetProtect was not given LLIN status based on data from six countries, two WHO sponsored studies and four studies sponsored by the manufacturer [4, 5]. Mean pHI after three years of deployment was 574 in Cambodia (pHI for reference net PermaNet 2.0 = 443, p = non-significant) and 78 in Ghana. NetProtect did not meet WHO bio-efficacy criteria based on the two WHO sponsored studies with a mean of 65% nets passing the combined cone and tunnel test. In the manufacturer-sponsored studies included in the WHOPES reports, 100% of the NetProtect passed bio-efficacy after 3 years in Kenya [6]. The Centers for Disease Control (CDC) conducted three evaluations of NetProtect included in WHOPES reports. The first study was conducted in Kenya demonstrated that both NetProtect and PermaNet 2.0 had a median survival of 2.5 years although no details of bio-efficacy or holed surface area were given [4]. In the second report [5] a study conducted in Kenya reported with 82% of NetProtect passing WHO bioassay criteria and median hole, surface area of 278 (0–4223) after 3 years of deployment. In Malawi, NetProtect showed 100% and median hole surface area of 188 (30.8–792) after 3 years. Using combined cone and tunnel test data from the WHO sponsored studies (N=2), 66% of nets passed bio-efficacy criteria and nets had a mean pHI of 326 (95% CI 0-3,477) at year three, thus the majority nets were in “serviceable” condition after 3 years.
***Review of all available data*:** Including data from peer reviewed publications in addition to the WHO sponsored studies (N=5), pooled proportion of nets passing bio efficacy criteria at 36 months was 81% and mean (95% CI) pHI was 300 (64-535).

Comparison of durability data to estimates generated in the current trial
Using the combined data from WHO and peer reviewed publications, the net durability data agrees with the data in the current study for estimates of bio-efficacy and fabric integrity after three years of operational use. The proportions of nets passing WHO bio-efficacy criteria was above 80% for NetProtect and PermaNet 2.0 and slightly below 80% for Olyset. NetProtect and PermaNet had similar fabric integrity with estimates within the serviceable range. Olyset showed lower fabric integrity relative to the other two brands in both the current study and the pooled analysis .

Summary of bio-efficacy criteria for NetProtect, Olyset and PermaNet 2.0 from a systematic review of the literature between 1990 and 2020 compared to the findings of the current trial.
[1] Median pHI used as data are overdispersed.
Data not included in calculating average proportionate hole index (pHI) and proportion passing bio-efficacy criteria because *data are included in WHOPES 2013, †average from WHOPES reports used, ‡ data at three-year time point not available.
§Data converted to mean pHI from data in original reports using weighting for small (1), medium (23) and large (196) hole sizes.

Reference	Net Brand	Location	Years of study	Follow up (years)	Sample size (nets)	Attrition (%)	Passed bioefficacy criteria (%)	Service-able (%)	Mean (median[1]) pHI
Current study	**NetProtect**	***Tanzania***	***2013-6***	***3***	***3,550***	***46***	***96***	***40***	***246 (29-1030)[1]***
Review		**Pooled average**		**3**			**83**		**300 (64-535)**
Odhiambo 2013 [6]		Kenya	2007-10	3	34	16	100		381
Van Roey 2014[7]*		Cambodia	2009-13	3	305	42	71		68[1]
WHOPES 2013 [4]		Cambodia	2009-13	3	305	40	69		574
WHOPES 2013 [4]		Ghana	2009-13	3		32	62		78
WHOPES 2014 [5]		Kenya	2009-13	3	663	29	82		278
WHOPES 2014 [5]		Malawi	2009-13	3	670	46	100		188
Current study	**Olyset**	***Tanzania***	***2013-16***	***3***	***3,529***	***55***	***75***	***27***	***564 (78-1044)[1]***
Review		**Pooled average**					**72**		**901 (143-1659)**
Dev 2016 [8]		India	2011-14	3	105	1		100	75
Dutta 2014 [9]		Vanauatu	2007-10	3	101			92	68
Massue 2016 [10]		Tanzania	2013	3	200	35	100	61	279
Morgan 2015 [11]		Mozambique	2008-11	3	47	21			3648
Toe 2019 [12]		Burkina Faso	2014-17	3	376	88	58	12	
WHOPES 2009 [3]		Benin	2008	3	10		70		1246
WHOPES 2009 [3]		Burkina Faso	2008	3	10		100		877
WHOPES 2009 [3]		Burundi	2008	3	10		30		1316
WHOPES 2009 [3]		Cote d'Ivoire	2008	3	10		100		399
WHOPES 2009 [3]		Niger	2008	3	9		60		566
WHOPES 2009 [3]		Togo	2008	3	10		60		536
WHOPES 2009 [3]		**WHOPES Average†**	**2008**	**3**	**59**		**71**		**823**

Reference	Net Brand	Location	Years of study	Follow up (years)	Sample size (nets)	Attrition (%)	Passed bioefficacy criteria (%)	Service-able (%)	Mean (median[1]) pHI
Ahogni 2020[‡] [13]	Olyset[‡]	Benin	2017-18	1	270	33		66	614
Allan 2012[‡] [14]		Chad	2007-09	1	293			61	76
Gnanguenon 2014[‡] [15]		Benin	2011-13	1.5	2002	58		69	859
Lindblade 2005[‡] [16]		Kenya	2002-04	2	49	80			
Mejia 2013 [17]		Kenya	2010	5	278	18		61	849
Tami 2004 [18]		Tanzania	1994-2002	7	7	0	100		
Current study	**PermaNet 2.0**	***Tanzania***	***2013-16***	***3***	***3,519***	***42***	***85***	***30***	***295 (41-1054)[1]***
**Review**		**Pooled average**		**3**			**84**		**380 (90-669)**
Dev 2016 [8]		India	2011-15	3	204	1		100	129
Killian 2008 [19]		Uganda	2000-05	3	260		100		
Morgan 2015 [11]		Mozambique	2008-12	3	86	21			834
Van Roey 2014 [7]*		Cambodia	2009-14	3	309		85		127[1]
WHOPES 2008 [20]		Angola	2007-08	3	12		100		148
WHOPES 2008 [20]		Ghana	2007-08	3	12		75		40
WHOPES 2008 [20]		Madagascar	2007-08	3	12		50		664
WHOPES 2008 [20]		Togo	2007-08	3	12		83		232
WHOPES 2008 [20]		Zambia	2007-08	3	12		92		862
WHOPES 2008 [20]		**WHOPES Average[†]**	**2007-08**	**3**	**60**		**80**		**389**
WHOPES 2013 [4]		Cambodia	2009-13	3	309	40	87		443
Ahogni 2020[‡] [13]	PermaNet 2.0[‡]	Benin	2017-19	1	270	28		99	194
Allan 2012[‡] [14]		Chad	2007-10	1	422			92.3	262
Solomon 2018[‡] [21]		Ethiopia	2014-16	2	993	96			
Wills 2013[‡] [22]		Ethiopia	2007-10	2.5	200			70	353

References

1. WHOPES. Report of the twelfth WHOPES working group meeting : WHO/HQ, Geneva, 9-11 December 2008 : review of Bioflash GR, Permanet 2.0, Permenet 2.5, Permanet 3.0, Lambda-cyhalothrin LN. Geneva: World Health Organization, 2009.
2. WHO. Vector Control Technical Expert Group Report to MPAC September 2013 Estimating functional survival of long-lasting insecticidal nets from field data. Geneva: World Health Organisation, 2013.
3. WHOPES. Report of the thirteenth WHOPES working group meeting : WHO/HQ, Geneva, 28-30 July 2009 : review of Olyset LN, Dawaplus 2.0 LN, Tianjin Yorkool LN. WHO/HTM/NTD/WHOPES/2009.5. Geneva: WHO Pesticide Evaluation Scheme, 2009.
4. WHOPES. Report Of The Sixteenth WHOPES Working Group Meeting Geneva: World Health Organisation Pesticide Evaluation Scheme, 2013.
5. WHOPES. Report Of The Seventeenth WHOPES Working Group Meeting Geneva: World Health Organisation Pesticide Evaluation Scheme, 2014.
6. Odhiambo MT, Skovmand O, Vulule JM, Kokwaro ED. Evaluation of polyethylene-based long lasting treated bed net netprotect on anopheles mosquitoes, malaria incidence, and net longivity in Western kenya. J Trop Med. 2013;2013:563957. Epub 2013/11/07. doi: 10.1155/2013/563957. PubMed PMID: 24194770; PubMed Central PMCID: PMCPMC3806382.
7. Van Roey K, Sovannaroth S, Sochantha T, Touch MS, Pigeon O, Sluydts V, et al. A phase III trial to evaluate the efficacy, fabric integrity and community acceptance of Netprotect using a recommended long-lasting insecticidal net as positive control. Malar J. 2014;13:256. Epub 2014/07/08. doi: 10.1186/1475-2875-13-256. PubMed PMID: 24998677; PubMed Central PMCID: PMCPMC4105388.
8. Dev V, Barman K, Khound K. A cross-sectional study assessing the residual bio-efficacy and durability of field-distributed long-lasting insecticidal nets in malaria endemic ethnic communities of Assam, Northeast India. J Infect Public Health. 2016;9(3):298-307. Epub 2015/11/22. doi: 10.1016/j.jiph.2015.10.005. PubMed PMID: 26589658.
9. Dutta SN, Amon J, Iata H, Cooper RD, Russell TL. Long-term insecticidal activity and physical integrity of Olyset nets in Tafea Province, Vanuatu. J Med Entomol. 2014;51(1):164-9. Epub 2014/03/13. doi: 10.1603/me13143. PubMed PMID: 24605466.
10. Massue DJ, Moore SJ, Mageni ZD, Moore JD, Bradley J, Pigeon O, et al. Durability of Olyset campaign nets distributed between 2009 and 2011 in eight districts of Tanzania. Malar J. 2016;15(1):176. Epub 2016/03/20. doi: 10.1186/s12936-016-1225-6. PubMed PMID: 26993981; PubMed Central PMCID: PMCPMC4797150.
11. Morgan J, Abilio AP, do Rosario Pondja M, Marrenjo D, Luciano J, Fernandes G, et al. Physical durability of two types of long-lasting insecticidal nets (LLINs) three years after a mass LLIN distribution campaign in Mozambique, 2008-2011. Am J Trop Med Hyg. 2015;92(2):286-93. Epub 2014/11/19. doi: 10.4269/ajtmh.14-0023. PubMed PMID: 25404079; PubMed Central PMCID: PMCPMC4347331.
12. Toe KH, Mechan F, Tangena JA, Morris M, Solino J, Tchicaya EF, et al. Assessing the impact of the addition of pyriproxyfen on the durability of permethrin-treated bed nets in Burkina Faso: a compound-randomized controlled trial. Mal J. 2019;18(383).
13. Ahogni IB, Salako AS, Akinro B, Sovi A, Gnanguenon V, Azondekon R, et al. Physical integrity and survivorship of long-lasting insecticidal nets distributed to households of the same socio-cultural community in Benin, West Africa. Malaria Journal. 2020;19(1):58. doi: 10.1186/s12936-020-3138-7.
14. Allan R, O'Reilly L, Gilbos V, Kilian A. An Observational Study of Material Durability of Three World Health Organization-Recommended Long-Lasting Insecticidal Nets in Eastern Chad. Am J Trop Med Hyg. 2012;87(3):407-11.
15. Gnanguenon V, Azondekon R, Oke-Agbo F, Beach R, Akogbeto M. Durability assessment results suggest a serviceable life of two, rather than three, years for the current long-lasting insecticidal (mosquito) net (LLIN) intervention in Benin. BMC Infect Dis. 2014;14:69. Epub 2014/02/11. doi: 10.1186/1471-2334-14-69. PubMed PMID: 24507444; PubMed Central PMCID: PMCPMC3925016.
16. Lindblade KA, Dotson E, Hawley WA, Bayoh N, Williamson J, Mount D, et al. Evaluation of long-lasting insecticidal nets after 2 years of household use. Trop Med Int Health. 2005;10(11):1141-50. Epub 2005/11/03. doi: 10.1111/j.1365-3156.2005.01501.x. PubMed PMID: 16262739.
17. Mejia P, Teklehaimanot HD, Tesfaye Y, Teklehaimanot A. Physical condition of Olyset(R) nets after five years of utilization in rural western Kenya. Malar J. 2013;12:158. Epub 2013/05/15. doi: 10.1186/1475-2875-12-158. PubMed PMID: 23663421; PubMed Central PMCID: PMCPMC3734157.
18. Tami A, Mubyazi G, Talbert A, Mshinda H, Duchon S, Lengeler C. Evaluation of Olyset insecticide-treated nets distributed seven years previously in Tanzania. Malar J. 2004;3:19. PubMed PMID: 15225349.
19. Kilian A, Byamukama W, Pigeon O, Atieli F, Duchon S, Phan C. Long-term field performance of a polyester-based long-lasting insecticidal mosquito net in rural Uganda. Malar J. 2008;7:49. Epub 2008/03/22. doi: 10.1186/1475-2875-7-49. PubMed PMID: 18355408; PubMed Central PMCID: PMCPMC2330059.
20. WHOPES. Report of the twelfth WHOPES working group meeting : WHO/HQ, Geneva, 9-11 December 2008 : review of Bioflash GR, PermaNet 2.0, PermaNet 2.5, PermaNet 3.0, Lambda-cyhalothrin LN. Geneva: World Health Organization, 2008.

21. Solomon T, Loha E, Deressa W, Balkew M, Gari T, Overgaard HJ, et al. Bed nets used to protect against malaria do not last long in a semi-arid area of Ethiopia: a cohort study. Malar J. 2018;17(1):239. Epub 2018/06/22. doi: 10.1186/s12936-018-2391-5. PubMed PMID: 29925371.
22. Wills AB, Smith SC, Anshebo GY, Graves PM, Endeshaw T, Shargie EB, et al. Physical durability of PermaNet 2.0 long-lasting insecticidal nets over three to 32 months of use in Ethiopia. Malar J. 2013;12:242. Epub 2013/07/17. doi: 10.1186/1475-2875-12-242. PubMed PMID: 23855778; PubMed Central PMCID: PMCPMC3733833.
