# Supplementary material for: Comparative functional survival and equivalent annual cost of 3 long-lasting insecticidal net (LLIN) products in Tanzania: A randomised trial with 3-year follow up
Source: PLoS Med. 2020 Sep 18;17(9):e1003248. doi: 10.1371/journal.pmed.1003248 (PMC7500675; doi:10.1371/journal.pmed.1003248)
Supplement: Attachment — Submitted filename: PMEDICINE-D-19-02821 response to reviewers_.docx [file pmed.1003248.s013.docx]

***AUTHOR RESPONSE: We thank the editor and the reviewers for their astute and helpful feedback. We have addressed each of the comments in turn below.***

Requests from the editors:

1- I think the title can be shortened: “Comparative functional survival and equivalent annual cost of three long lasting insecticidal net products in Tanzania: a three-year prospective cohort study”

***AUTHOR RESPONSE: This has been shortened***

2- Thank you for listing the authors’ competing interests. Please list, either in the CI statement or in a supplemental file if the list is very long, the manufacturers that the authors work with.

***AUTHOR RESPONSE: This has been added***

3- Thank you for agreeing to make the data available and providing the link for data access. Please also provide any accession number(s) or search terms a researcher will need to obtain access to this specific dataset.

***AUTHOR RESPONSE: This has been added***

Access to the data sets must be requested from NSD using this order form: <https://nsd.no/nsd/english/order.html> and referring to the project ID no. NSD2695 and project name “The useful life of bednets for malaria control in Tanzania: Attrition, Bioefficacy, Chemistry, Durability and insecticide Resistance”, project no. 220757

4- The Abstract Background is a bit long, please shorten by about half.

***AUTHOR RESPONSE: This has been reduced from 182 words to 107 words***

5- Please amend “We conducted the largest prospective study of LLIN…” with “to our knowledge” or similar.

***AUTHOR RESPONSE: This has been removed from the abstract***

6- Please include full date ranges for the study in the Abstract Methods and Findings section.

***AUTHOR RESPONSE: This has been added***

7- In the Abstract Methods and Findings section, please include both 95% CIs and p values for the study outcomes, including the cost estimates.

***AUTHOR RESPONSE: This had been added***

8- Please delete the final sentence of the Abstract Methods and Findings section (“However, the setting is more likely to...”).

***AUTHOR RESPONSE: This was deleted***

9- Please preface the Abstract Conclusions with: “These results suggest…” or similar.

***AUTHOR RESPONSE: This was amended***

10- At this stage, we ask that you include a short, non-technical Author Summary of your research to make findings accessible to a wide audience that includes both scientists and non-scientists. The Author Summary should immediately follow the Abstract in your revised manuscript. This text is subject to editorial change and should be distinct from the scientific abstract. Please see our author guidelines for more information:https://journals.plos.org/plosmedicine/s/revising-your-manuscript#loc-author-summary

***AUTHOR RESPONSE: Author summary has been added***

11- Thank you for providing the STROBE checklist. Please replace the line numbers with paragraph numbers per section (e.g. "Methods, paragraph 1"), since the line numbers of the final published paper may be different from the page numbers in the current manuscript.

***AUTHOR RESPONSE: This has been updated***

12- Please remove the registered trademark symbols throughout the manuscript.

***AUTHOR RESPONSE: These have been removed***

13- S1 Table could be moved to the main text.

***AUTHOR RESPONSE: This has been moved to the main text and is now table 1***

14- Please begin the Discussion with a brief summary of what was done, before getting to the findings and interpretations.

***AUTHOR RESPONSE: A summary was added***

15- The current first sentence of the Discussion would be better stated: “The findings of this study demonstrate that there is considerable variability in the lifespan of pyrethroid-treated LLIN products.”

***AUTHOR RESPONSE: This suggestion has been added***

16- On line 317, perhaps “Our findings demonstrate that nets that are still in households…”

***AUTHOR RESPONSE: This suggestion has been added***

Comments from the reviewers:

Reviewer #1: Although at first glance, this study may not appear particularly novel, and the results do not yield any major surprises it is an important paper that raises some fundamental points that urgently need to be addressed by all involved with bednet manufacturer, procurement and distribution.

***AUTHOR RESPONSE: We thank the reviewer for their positive comments and are pleased they see the value of our study***

As the authors point out, long lasting insecticidal nets (LLINs) represent a huge market with billions having been distributed across Africa in the 21st century. To be classified as 'long lasting' these nets must last three years under operational use and the World Health Organisation have issued guidelines on how these nets should be evaluated. But such prospective studies are rarely conducted in practice and surprisingly little has been published on the effective life of LLINS, particularly considering the billions of dollars spent on these. This paper looks at this specific issue in a study across multiple districts in Tanzania. In this particular study they find that one brand of net in inferior compared to two further nets tested. This is an important result for Tanzania and arguably for other countries too; although some might argue that the results will be context specific, as the authors point out it seems reasonable to expect the relative performance to remain.

The paper is weakened by not testing any of the net samples on resistant mosquitoes; this point is acknowledged by the authors but it cannot be overlooked. The vast majority of Anopheles mosquitoes in Tanzania, and across Africa, will have some level of resistance to pyrethroid insecticides. When nets are initially distributed they may still have sufficient insecticide to kill both susceptible and resistant mosquitoes but as the amount of active ingredient (AI) decreases (as observed in this study where two of the products have lost a fifth or a third of the AI within 10 months of use) it is possible that resistant mosquitoes will survive a short exposure to these nets and hence the operational efficacy of the nets will be compromised. The failure to measure the bioefficacy against resistant mosquitoes is a major limitation, particularly given it may have altered the key conclusions of the paper.

***AUTHOR RESPONSE: We acknowledge in the introduction and in the discussion that insecticide resistance is another a growing threat – in addition to short net-life - to the protection given by LLINs. We also acknowledge that holes and insecticide residuality and resistance interact in a complex manner to determine effectiveness/ protection. But the evolutionary process that causes resistance is quite distinct from the physical processes that cause fabric damage, decline in insecticidal activity and loss of nets. Therefore, it is sensible to study the latter independent of the former, and doing so does not imply anything about the relative importance of resistance vs durability.***

***The focus of this paper is LLIN durability, and for this reason we followed WHO guidelines for measurement of durability to maximise the generalisability of the findings. The WHO guidelines advise the use of pyrethroid susceptible mosquito strains for the testing of pyrethroid only nets which allows the detection of declining bioefficacy in the nets. The reviewer makes an important point that it is possible that resistant mosquitoes will survive a short exposure to these nets and hence the operational efficacy of the nets will be further compromised.***

***However, we do not feel that the addition of a resistant strain to the bioassays would alter the key conclusions of the paper because 1) we are measuring the relative functional efficacy of different pyrethroid nets, 2) the majority of the loss of functional life was as a result of declining fabric integrity that resulted in domestic users choosing to discard their nets.***

***New product classes of LLINs with novel active ingredients for insecticide resistance management are becoming available, but they remain susceptible to the same forces of physical disintegration, being discarded and losing insecticidal activity as pyrethroid nets. The paper makes the case that for urgent consideration of functional survival of LLINs that includes the attrition and loss of fabric integrity of LLINs is required precisely because new (more expensive) products are coming to the market to address insecticide resistance. Consideration of value for money among institutional buyers has never been more critical.***

I believe the paper could be strengthened in the following ways

1. It is essential to provide greater clarity how these nets were originally classified as LLINs. In the introduction (lines 27-29) the process is briefly described but, to evaluate the results of this manuscript, it is important that readers are aware of the specific evaluations that these three net products have undergone previously. This is important to understand if the current results are an anomaly or agree with previous studies. In the discussion this is mentioned again (line 275-277) but again no information on the previous evidence warranting the WHO classification of LLIN is provided. I would like to see a supplementary file, providing a summary of the durability data used by WHO to evaluate the three net types included in the current study, plus any subsequent published data on the durability of these nets (the authors don't even mention whether these results agree with their own retroactive study in Tanzania (Ref 26)). Furthermore, a fuller discussion of the extent to which WHO PQ are following these guidelines in their listings of new nets (particularly the new LLINs which contain more than one insecticide) must be included in the discussion. I agree with the authors conclusions that durability is too often overlooked but the extent to which this is happening, and the potential consequences of this could be more clearly stated.

***AUTHOR RESPONSE: This important point has been added to the introduction (paragraphs 3-5) and discussion paragraph 4 to clarify this***

***A literature review was performed and added as Supplementary material S1 Table. The results of the current study agree with those of earlier studies.***

***Results from other studies have been added to the discussion paragraph 1 and discussion paragraph 1.***

2. If I remember correctly the weave design of Olyset changed at some point in the last six years; given the poor fabric integrity for Olyset reported it is critical to clarify if nets used here are those currently on the market.

***AUTHOR RESPONSE: This is indeed a critical point. We thank the reviewer for highlighting the omission of this detail. The Olyset nets in this study did indeed use the newer weave that the reviewer mentions. This has been added to the text Methods Paragraph 1***

3. Methods: The sampling strategy requires clarification

a. Were the three nets sampled per household pre-selected before entering (as opposed to convenience sampling on arrival)

***AUTHOR RESPONSE: This has been clarified in methods paragraphs 2 and 3 with the sentences:***

***Physical integrity of LLINs was measured on a maximum of three nets per household by counting the number, location and size of holes [12, 23]. In households with more than three nets, a random sample was selected from the master list for that household.***

***In addition to the data collected as part of the household surveys, at each time point 48 LLINs from each brand were randomly pre-selected from the master list using a random number generator and returned to the laboratory***

b. When nets were sampled for bioeffiacy assays, was the net replaced? If so was the replacement clearly labelled to avoid being sampled in subsequent rounds?

***AUTHOR RESPONSE: Methods paragraph 3 text added: Households received new nets to replace those removed for destructive sampling. Once a house had been sampled, it was eliminated from the master list to prevent confounding of results.***

4. Figure 2 legend should make clear what the denominator is (nets remaining in household at time of survey?) and indicate sample size for each row. This figure gives a somewhat misleading picture as it ignores nets that have been removed from households, many of which might be too torn. An additional (or replacement) figure including nets lost to follow up would be informative.

***AUTHOR RESPONSE: The reviewer is correct that the denominator was nets remaining in household at time of survey. We have now included nets that were lost to attrition as the reviewer suggests and included denominators in the figure legend.***

5. Figure 3 requires sample sizes. Please also change y scale from 0-100 as the current graph presents a misleading picture on the difference between the nets.

***AUTHOR RESPONSE: We have added sample sizes. We prefer to leave the y axes as they are. If the axes are changed as the reviewer suggests the differences between the net types, which we believe are important, will not be possible to see. The y axes are clearly labelled so we do not think they will be misleading to the reader.***

6. The results section 'Active ingredient content' is confusing for non-chemists. What is the biological significance of the R-alpha content? I assume this is not bioactive? The very high rate of loss of AI in two of the nets is not discussed further. This does not appear to relate to bioefficacy against susceptible mosquitoes but as the study did not test resistant strains, it is not possible to state whether this loss of insecticide would affect their operational efficacy.

***AUTHOR RESPONSE: The sentence was removed: Deltamethrin content lower than the target dose in NetProtect was explained by a high R-alpha isomer content (0.35 g/kg on average, 26% of the deltamethrin content), a non-relevant impurity of deltamethrin, which may be formed during the manufacturing process.***

***We also added a sentence: While this loss of insecticide did not negatively impact the bioefficacy of the nets against a pyrethroid resistant strain of mosquito, it is plausible that it would impact the efficacy of the nets against more resistant mosquitoes***

7. Discussion, line 282, states that attrition and physical integrity are the two factors that define functional survival of LLINs - again I would query whether this is the case if you considered pyrethroid resistance. Churcher et al, e-Life 2018 have shown how net durability is influenced by resistance. This need acknowledging

***AUTHOR RESPONSE: This reference has been added***

***Attrition and fabric integrity, the two factors that define physical survival of LLINs [31] [32], differed significantly between the three net products.***

8. There was a national distribution of LLINs in Tanzania from 2015-2017, was the current study area included and, if so, how did this impact on the current study?

***AUTHOR RESPONSE: The distribution occurred after the study was completed in all but one study district. The impact of the distribution has been explored in a separate publication (Mboma et al in preparation) and was limited because most houses with ABCDR nets continued to use them.***

9. Discussion, line 253, states that median functional lifespan was closer to 2 years. However, Table 2 states the median life span to be 2.0, 2.5 and 2.6 for the three nets - is this really closer to 2 years? A more accurate statement could be obtained if the lifespan of the pooled analysis was presented. If it is closer to 2.5 years rather than 2 years, state this. Six months is important in this context!

***AUTHOR RESPONSE: Thank you – good point. We have changed this to less than 3 years***

10. The analysis of the IACT tests mentions adjustment for physical condition. Please include further explanation of how this was performed in the data analysis section.

***AUTHOR RESPONSE: We have added the following line to the statistical analysis section to explain what we did: “A further analysis was conducted to test for differences in mortality between net brands in the IACT test based on net condition, in which net condition (defined above) was adjusted for as a fixed effect.”***

11. Discussion, line 299 'most damage to the nets is accumulated on the bottom section of the nets….'. Is this from this study? I couldn't find any information on the location of holes from the supplementary data tables. If not from this study a reference must be given.

***AUTHOR RESPONSE: This is data from the current study but is being written up for a different publication. We have therefore stated “data not shown”***

Reviewer #2: Statistical review

This paper reports a randomised study comparing the longevity and laboratory-assessed efficacy of mosquito nets. The authors show there are significant differences between the different types in both of these characteristics as assessed by different outcomes.

I had some comments on the statistical methods and reporting, which I have provided below.

1. Title: I was not sure why the title refers to this as a prospective cohort study, but the rest of the paper refers to it as a RCT - I would recommend this is kept consistent.

***AUTHOR RESPONSE: This has been consistently referred to as a prospective cohort study***

2. Abstract and results - I think it would be useful if confidence interval in differences of net functional survival was given between groups together with CIs and p-values; currently unclear (at least in the abstract) if the differences here are significant or not.

***AUTHOR RESPONSE: We have added a p-value to the abstract in addition to the confidence intervals.***

3. I did not find it completely clear from the methods section which outcomes were assessed by which data source (i.e. the inspection of the house or the lab tests on the restricted number). From what I understand, attrition, physical integrity and functional survival come from the household inspections and biological efficacy/insecticide content from the lab tests - please clarify if this is wrong.

***AUTHOR RESPONSE: Yes, the reviewer understood correctly. In the statistical analysis section, it now states where data on each outcome came from.***

4. Line 110 "adjusted for control mortality" - I didn't follow what exactly is being adjusted for here.

***AUTHOR RESPONSE: We agree this was not clear. We have now explained that for the WHO cone assay, data from assays with control mortality over 10% was not included in the analysis.***

5. Methods - how was missing data handled in the analysis?

***AUTHOR RESPONSE: For attrition and functional survival, survival analysis methods were used. To do this one must assume of non-informative censoring on losses to follow up, and we made this standard assumption. The lab tests on bioefficacy were done in a controlled environment in which there were no missing data.***

6. Statistical/economic analysis methods - was a pre-specified analysis plan developed for this analysis? It should be provided if so.

***AUTHOR RESPONSE: No analysis plan was written for the study, but study outcomes were prespecified in a published protocol (Lorenz et al 2014*** [*https://bmcpublichealth.biomedcentral.com/track/pdf/10.1186/1471-2458-14-1266*](https://bmcpublichealth.biomedcentral.com/track/pdf/10.1186/1471-2458-14-1266)*).*

7. Page 10 line 159: is the p-value here testing if there's any difference between the three types of net? If not, could it be clarified what the p-value is testing?

***AUTHOR RESPONSE: Yes, the p-value is testing if there's any difference between the three types of net.***

8. Table 3 - is there any significant difference between permanet and netprotect here?

***AUTHOR RESPONSE: Yes, the difference was significant. We have added this to the table (now labelled Table 4).***

9. Table 4 - similarly here, it doesn't appear that permanet or netprotect would be significantly different?

***AUTHOR RESPONSE: No, there were no significant differences between permanet and netprotect. We have added this information to the table (now labelled Table 5).***

James Wason

Reviewer #3: The manuscript presented by Lorenz et al. on the comparative functional survival and equivalent annual cost of three long-lasting insecticidal nets (LLIN) is clearly the best systematic assessment of LLIN durability to-date. The issue of how long LLINs last in the field is rapidly becoming the top priority topic for LLIN programmess, allongside insecticide resistance. Given that public entities spend in excess of USD 250 millions per year on LLIN deployment, and given that this represents our primary malaria prevention measure in most malaria-endemic settings, the importance of any major factor affecting the performance of LLINs can not be over-emphasized.

The authors present here a well designed and well powered study comparing directly and in a randomized design the three brands of LLINs which largely dominate the market. These results are eagerly awaited by the malaria community and it is important to publish them rapidly. In addition to these important results, this manuscript also presents a number of new approaches to quantifying the issue of uselife of nets (or validates their use on a large scale), as well as a methodologically appropriate way to estimate the cost versus the expected effects of a product on the basis of its field characteristics. As a result of the above, I consider this a manuscript of high public health importance.

***AUTHOR RESPONSE: We thank the reviewer for their positive comments and are pleased they see the importance of this paper.***

The methodology of this work is largely sound and well implemented, and the results are well analyzed and described. The article is also well written and nearly all required information is there. I have therefore only one issue for which clarification is required.

1. Economic analysis (p 9 and 11). The authors have done the economic analysis on the basis of a uniform net price of USD 3. They should better justify their decision to take this price value for two reasons: (1) most factory prices of nets are lower than that amount these days, and (2) This price does not include the distribution costs as well as all the non-commodity costs associated with net distribution, such as Behaviour Change components, etc. The distribution costs (planning, census, transport, etc.) are particularly important to consider in this work because a net lasting longer would allow to lengthen the time interval between distributions, and that part of the total LLIN-in-the-field cost would be reduced proportionally. I appreciate that the authors have probably not taken this into account in their calculations, but they should at least discuss this point and outline what the wider implications are of an expended durability of LLINs in the field.

***AUTHOR RESPONSE: We agree that current standard LLINs are now available at factor or CIF prices below 3 USD but this is a relatively close approximation and the qualitative results presented here are agnostic to the specific price as they are relative, we chose 3 USD because it is close to the current prices and a round number which is easy to communicate. New nets with dual AI and PBO incorporated are significantly more extensive at this time so we think using a lower number than three would be more misleading.***

***We also agree that one of the potential advantages to longer lasting nets would be the potential to lengthen the time between campaigns, or to delivery fewer nets in a continuous distribution campaigns thus leading to a cost savings. However, the decision to lengthen time between campaigns would require a weighing of changes in coverage, cost and timing of campaign and would induce non-linearities in the decision framework. Since these questions are much more complex and dependent on distribution modality (e.g. campaigns vs. ANC/EPI) as well as the effectiveness of the nets and would require a full manuscript to describe, we have focused only on the EAC (or cost per useful life) we have added to the discussion more text to make this rationale clear.***

Finally, the limitations of the study are well described, once the points above are included.
